# Embodiment and Humiliation Moderation of Neural Responses to Others' Suffering in Female Submissive BDSM Practitioners

**DOI:** 10.3389/fnins.2018.00463

**Published:** 2018-07-09

**Authors:** Siyang Luo, Xiao Zhang

**Affiliations:** ^1^Guangdong Key Laboratory of Social Cognitive Neuroscience and Mental Health and Guangdong Provincial Key Laboratory of Brain Function and Disease, Department of Psychology, Sun Yat-sen University, Guangzhou, China; ^2^Institute for Economic and Social Research, Jinan University, Guangzhou, China

**Keywords:** BDSM, empathy, physical restriction, embody, humiliation

## Abstract

Giving and receiving pain are common in the practice of BDSM (bondage-discipline, dominance-submission, and sadism-masochism). Playing a submissive role during BDSM practice weakens both the behavioral and neural empathic responses of female individuals to others' suffering, suggesting that long-term BDSM experience affects BDSM practitioners' empathic ability. This study further investigates whether physical restriction during BDSM practice also modulates individuals' neural responses to others' suffering. We measured neural responses to others' suffering by recording event-related potentials (ERPs) in female submissives while they viewed painful and neutral expressions in sexual sadistic/general social contexts under ball gag Blocking and Relaxed conditions. The neural responses recorded during 92–112 ms (N1), 132–172 ms (P2), 200–340 ms (N2), early late positive potential (LPP, 400–600 ms), and late LPP (700–1,000 ms) were included in the analyses. Compared to the relaxed condition, when a ball gag was used to prevent facial muscle movement and facial mimicry, the N1, early LPP, and late LPP responses neural responses to others' suffering were inhibited. The moderation effect of ball gag blocking on the N1 and early LPP amplitudes was positively correlated with the subjective feelings of facial muscle stillness, and the blocking moderation effect on the late LPP amplitudes was positively correlated with subjective feelings of humiliation. This study is the first neuropsychological investigation of the transient BDSM-related physical restriction effects on BDSM practitioners. These findings suggest that physical restriction (via a ball gag) during BDSM practices increases the wearer's facial muscle stillness and sense of humiliation. This physical restriction inhibits both early automatic responses and late controlled processes in response to the suffering of others.

## Introduction

BDSM, which is a combination of the abbreviations B/D (bondage and discipline), D/S (dominance and submission), and S/M (sadism and masochism) (Fedoroff, 2008), is common in all social segments and is practiced by both homosexual and heterosexual individuals. BDSM includes many types of sexual practices or roleplaying, such as bondage, physical restriction, punishment, and power exchange (Alison et al., 2001; Sandnabba et al., 2002; Connolly, 2006; Wismeijer and van Assen, 2013). In addition, many BDSM practices involve the exchange of physical or emotional pain (Wismeijer and van Assen, 2013). For example, Breyer et al. (2010) found that more than half of bisexual women have been restrained for pleasure, and 36% of bisexual women have received pain for pleasure. Frequent exposure to pain-inflicting situations not only affects individuals' pain perception but also influences their perception of the pain of others (Cheng et al., 2007).

These subjective responses to other people's suffering, also known as empathy for pain (Batson, 2011), play a key role in prosocial behavior and function abnormally in multiple disorders, such as psychopathy and borderline personality disorder (Soderstrom, 2003; de Waal, 2008; Harari et al., 2010). During the prior decade, the underlying neural mechanisms of empathy have been extensively studied using neuroimaging techniques. Previous studies using functional magnetic resonance imaging (fMRI) have shown that viewing others' suffering activates the neural circuit “pain matrix,” which consists of the anterior cingulate cortex (ACC), bilateral anterior insula, supplementary motor area (SMA), and second somatosensory cortex (SII) (Singer et al., 2004; Jackson et al., 2005; Luo et al., 2014, 2015b). Event-related potential (ERP) studies have also revealed that compared to non-painful stimuli, the perception of painful stimuli applied to others modulates the amplitudes of both the early and late ERP components in the frontocentral and centroparietal areas (Decety et al., 2010; Sheng and Han, 2012; Han et al., 2016).

To date, increasing studies have shown that empathic neural responses to others' suffering are strongly modulated by social and biological factors, such as personal experiences (Cheng et al., 2007), bodily state (Han et al., 2016), affective link (Singer et al., 2006), cultural background, and genetic makeup (Zuo and Han, 2013; Luo et al., 2015a,b). For example, physicians show less empathy-related pain activity in the AI and ACC than healthy controls, suggesting that empathic brain responses are reduced in empathizers who are frequently exposed to pain-inflicting situations (Cheng et al., 2007). We previously examined the empathy trait and empathic responses to others' suffering in BDSM practitioners. Practitioners play various roles during BDSM practice, such as the dominant role (Dom; the person who exerts control), the submissive role (Sub; the person who gives up control), or both roles depending on the situation (Switches) (Wismeijer and van Assen, 2013). We found that female submissives showed lower trait empathy scores and subjective pain intensity ratings than the controls; however, the empathy trait in male BDSM practitioners did not significantly differ from that in the control group (Luo and Zhang, 2017). The electroencephalogram (EEG) recordings further revealed that the differential amplitudes in response to painful and neutral expressions in the frontal N1 (92–112 ms), frontal P2 (132–172 ms), and central late LPP (700–1,000 ms) were lower in the submissive group than those in the control group, suggesting that playing the submissive role during BDSM practice weakens female individuals' empathic responses to others' suffering at both the behavioral and neural levels. However, these studies mainly focused on the effect of long-term BDSM experience on practitioners' empathic ability, and knowledge regarding whether and how the transient physical restriction used in BDSM practice modulates individuals' neural responses to others' suffering is limited.

Previous researchers have proposed the concept of “embodying emotion,” which suggests that processing the emotional states of others involves a perceptual and somatovisceral embodiment of one's own emotions (Niedenthal, 2007). When participants were asked to hold a pen horizontally using both their teeth and lips to prevent facial muscle movement and facial mimicry, the frontal N1 response (100–120 ms) to painful expressions was significantly reduced (Han et al., 2016). Similarly, the ball gag, which is a device that is sometimes worn during sexual bondage and BDSM practice, may also prevent facial muscle movement and facial mimicry, thereby inhibiting neural responses to others' suffering. In addition to the N1 component, a long-latency neural response was observed after 300 ms over the central parietal regions; this response is usually called the LPP component or P3 component and is suggested to be related to empathy (Fan and Han, 2008; Han et al., 2008). Fan and Han (2008) revealed differential ERPs in response to painful and neutral stimuli over the central parietal regions at 380 ms after sensory stimulation. Subsequent research further found that the LPP component could be regulated by the participants' emotional states and social contexts (Fan and Han, 2008; Olofsson et al., 2008; Feng et al., 2012, 2014). For example, erotic pictures evoked enhanced P3 amplitudes (300–440) compared with other pictures. In addition, a ball gag can also increase the wearer's sense of helplessness during BDSM practice by rendering them unable to speak. For some people, gags have connotations of punishment and control and, thus, can be used as a form of humiliation or dehumanization (pony or animal play). According to our preliminary interviews with 19 BDSM practitioners (6 male, 13 female), 100% individuals report shame feeling and 79% individuals report dehumanization feeling (like an animal belong to Doms) when wearing a ball gag. These humiliation or dehumanization processes may also have an effect on the LPP responses to others' suffering.

In this study, we investigated whether wearing a ball gag modulates BDSM practitioners' neural responses to others' suffering by recording ERPs in BDSM practitioners under ball gag Blocking and Relaxed conditions. The neural responses to others' suffering were calculated by contrasting the responses to perceived painful stimuli with those to non-painful facial expressions, which is similar to previous studies (Sheng and Han, 2012; Luo et al., 2014; Han et al., 2016). Furthermore, we assessed whether the context of the painful expression (painful expression in a general context vs. painful expression in a sadistic context) moderated the neural responses to others' suffering. In contrast to a non-social context (e.g., Alex knocked the wall and hurt his knee), in common sexual sadistic contexts, the suffering of submissives is inflicted intentionally by another person (e.g., someone's body is beaten with a whip). These sadistic contexts have clear social implications that suggest that someone's painful experience was inflicted by a sadist and that these sadistic behaviors will continue. Furthermore, in the sadistic context, criminal sexual sadists lack the ability to empathize with the victims, which may lead to an increased likelihood of perpetrating instrumental violence (Kirsch and Becker, 2007). Therefore, the social context in which pain occurs may modulate the neural responses to the pain of others (Akitsuki and Decety, 2009). Thus, the neuroimaging results obtained in the current study allowed us to examine whether there were consistent differences in neural responses to others' suffering between the ball gag blocking condition and the relaxed condition across sexual sadistic and non-relevant general contexts and provided new insight into the neural underpinnings of empathy for pain during BDSM practices.

## Methods

### Participants

The BDSM practitioners who participated in this study were recruited from a previous BDSM practitioner sample in China (Luo and Zhang, 2017). The first criterion for choosing the BDSM practitioners was that their answers to the following two questions were “yes”: Do you like BDSM practices? Have you ever engaged in BDSM practices? We divided the BDSM practitioners into the dominant group (Doms), switch group (Switches), or submissive group (Subs) based on their subjective reports of their roles. Twenty-six female submissive individuals aged 18–30 years participated in this study as paid volunteers. This sample size is similar to that in a previous study (Han et al., 2016) and could detect a medium effect size for within-subject interactions (*f* = 0.25, alpha = 0.05, power = 0.80, correlation for repeated measures = 0.30). All participants reported experiencing at least one BDSM practice and performed only submissive roles during BDSM practice (not including switch). The exclusion criteria included self-reported medical or psychiatric illness and the use of medication. Before conducting the study, written informed consent was obtained from all participants, and this study was approved by the Institutional Review Board of the Department of Psychology of Sun Yat-Sen University.

### Stimuli and procedures

**Figure 1:**
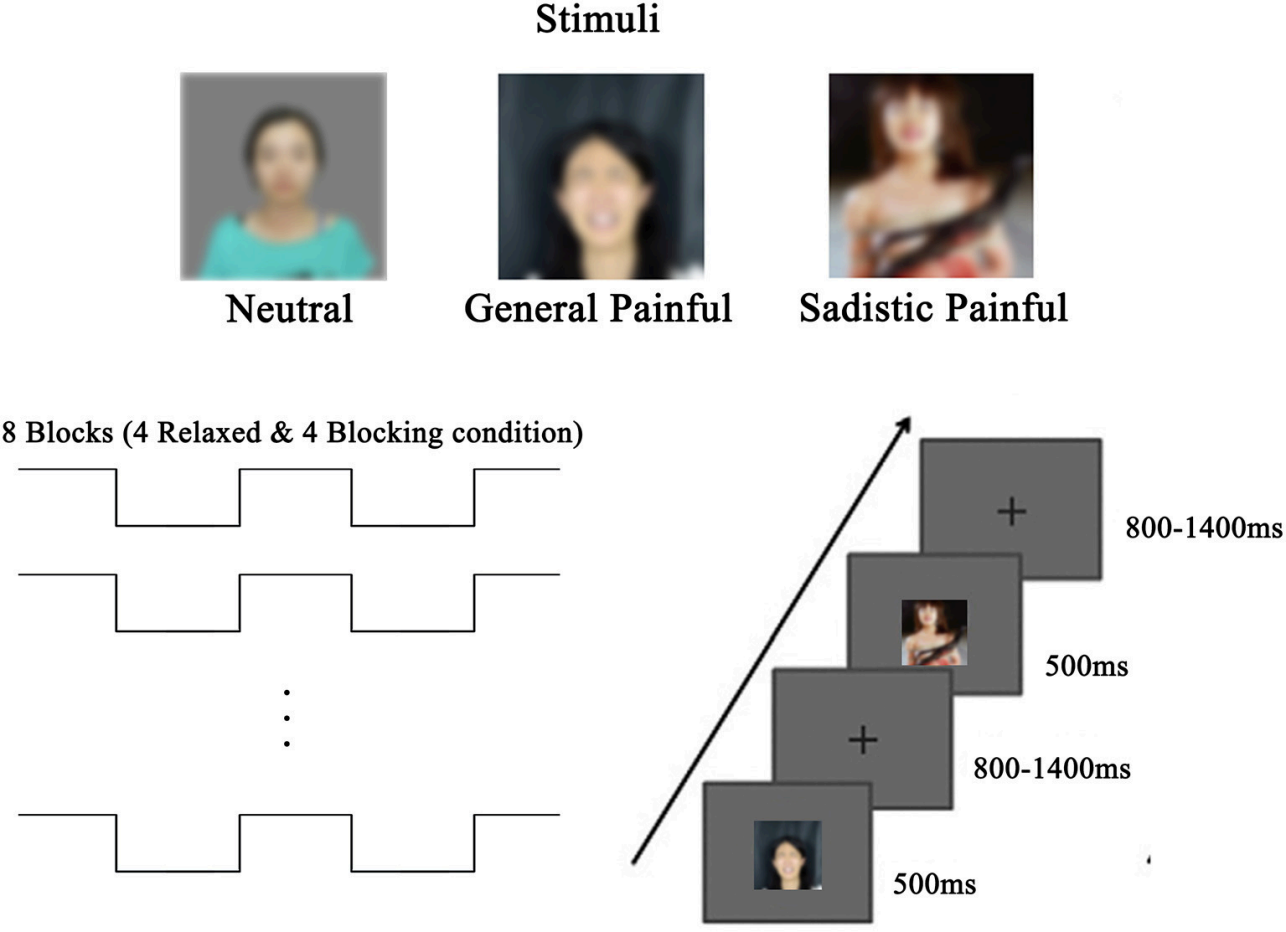
Experimental design and stimuli.

The stimuli used during the EEG recordings were the same as those used in a previous study (Luo and Zhang, 2017) and consisted of 48 digital photographs of female faces with neutral or painful expressions, including 16 neutral expressions (no painful or BDSM information involved), 16 painful expressions in general contexts (no BDSM information involved) and 16 painful expressions in sexual sadistic contexts (e.g., a female model showing a painful expression while being whipped, Figure 1). The current study adopted a within-subject design with blocking (ball gag blocking (blocking) vs. Relaxed) and expression (neutral/general painful/sadistic painful) as the independent variables. The participants completed 8 EEG blocks of 96 trials during the experiment. Each photo was presented twice in random order in each block. The participants were asked to hold a ball gag in their mouths in four of the blocks of trials, whereas there was nothing in their mouths during the other four blocks of trials. There was a 10-min break between the ball gag Blocking and Relaxed blocks of trials. The order of the blocking and relaxed conditions was counterbalanced across participants. During the EEG recordings, each face had a visual angle of 4.7° × 4.7° (width × height: 9.92 × 9.92 cm) at a viewing distance of 120 cm and was displayed in the center of a gray background for 500 ms. The interstimulus intervals consisted of a fixation cross with a duration that randomly varied between 800 and 1,400 ms. The participants judged the expression of each face (painful vs. neutral) and pressed a button using their right index and middle fingers during each trial.

After the EEG recordings, the participants were asked to rate the degree of facial muscle stiffness and subjective feelings of shame and sexual arousal during the blocking and relaxed blocks on a 9-point Likert scale.

### EEG recordings and analysis

The EEG recordings were performed using 64 scalp electrodes (based on the 10/20 system), and two electrodes were placed on the left and right mastoids. Eye blinks and vertical eye movements were monitored with electrodes located above and below the left eye. A horizontal electrooculogram was recorded from electrodes placed 1.5 cm lateral to the left and right external canthi. The EEG was amplified (band pass 0.01–100 Hz) and digitized at a sampling rate of 250 Hz. All data were re-referenced off-line to an average mastoid reference and filtered high frequency noise from muscle tension using a band-pass filter (0.1–40 Hz). To further exclude the confounding factors of short prestimulus interval and low frequency noise, we also used 500ms as the prestimulus interval and a band-pass filter (1-40 Hz) to analyse our data (see Supplementary Information). The ERPs from each condition were averaged separately off-line using an epoch beginning 200 ms before the stimulus onset and continued for 1,500 ms after the stimulus onset. Trials with excessive response errors or eye movements and muscle potentials exceeding ±50 μV at any electrode were excluded from the average. The baseline of the ERP measurements was the mean voltage of a 200 ms pre-stimulus interval, and the latency was measured relative to the stimulus onset. The mean amplitudes of each ERP component were calculated at electrodes selected from the frontal (Fz, F3, F4, F5, F6, FCz, FC3, FC4, FC5, and FC6), central (Cz, C1, C2, CPz, CP1, and CP2), parietal (Pz, P1, and P2), and occipito-temporal (PO7, PO8, P7, and P8) regions. Consistent with previous studies (Luo and Zhang, 2017; Luo et al., 2017), the ANOVAs of the mean ERP amplitudes recorded at the bilateral electrodes included hemisphere (electrode over the left vs. right hemisphere) as a within-subject variable.

Both voltage topography and standardized low-resolution brain electromagnetic tomography (sLORETA) (Pascual-Marqui, 2002) were used to estimate the potential sources of the empathic neural responses. sLORETA is a linear method for computing statistical maps from EEG data that reveals the locations of the underlying source processes and does not require a priori hypotheses regarding the field distribution of the active sources. We performed the analysis using sLORETA to assess the 3D current source of the neural activity that differed in the ERPs between the painful and neutral expressions. A boundary element model was first created with ~5,000 nodes from a realistic head model. Statistical nonparametric mapping was performed in a specific time window to estimate the source that differed in the ERPs between the painful and neutral expressions. The sLORETA time windows were consistent with the time windows used in the ERP analysis. The log of the F ratio and t values of the averages were used and considered at a significance level of 0.95.

## Results

The subjective feelings of facial muscle stiffness under the blocking condition were significantly higher than those under the relaxed condition [blocking: 7.27 ± 1.54; relaxed: 1.27 ± 0.53, *t*_(25)_ = 21.63, *p* < 0.001], indicating that the participants experienced increased muscle tension when they held a ball gag in their mouths compared to when they were able to move their facial muscles freely. The subjective ratings of pain intensity, unpleasantness, enjoyableness, and arousal in response to the neutral, general painful and sadistic painful stimuli were showed in Table 1. The subjective rating results of shame and sexual arousal during blocking and relaxed condition were showed in Table 2.

**Table 1:** Subjective ratings of pain intensity, unpleasantness, enjoyableness, and arousal in response to the neutral, general painful, and sadistic painful stimuli (Mean±SD).

**Variable**	**Neutral**	**General painful**	**Sadistic painful**
Pain intensity	1.61 ± 0.78	4.85 ± 1.55	5.44 ± 1.55
Unpleasantness	1.86 ± 1.11	3.22 ± 1.71	3.31 ± 1.66
Enjoyableness	4.01 ± 1.84	2.35 ± 0.94	6.22 ± 1.32
Arousal	2.07 ± 1.45	1.88 ± 0.91	5.39 ± 1.68

**Table 2:** Subjective ratings of facial muscle stiffness and subjective feelings of shame and sexual arousal under the blocking and relaxed conditions (Mean ± SD).

**Variable**	**Blocked**	**Relaxed**
Muscle stiffness	7.27 ± 1.54	1.35 ± 0.69
Shame	4.46 ± 2.10	2.31 ± 1.35
Sexual arousal	3.73 ± 1.54	2.50 ± 1.24

**Figure 2:**
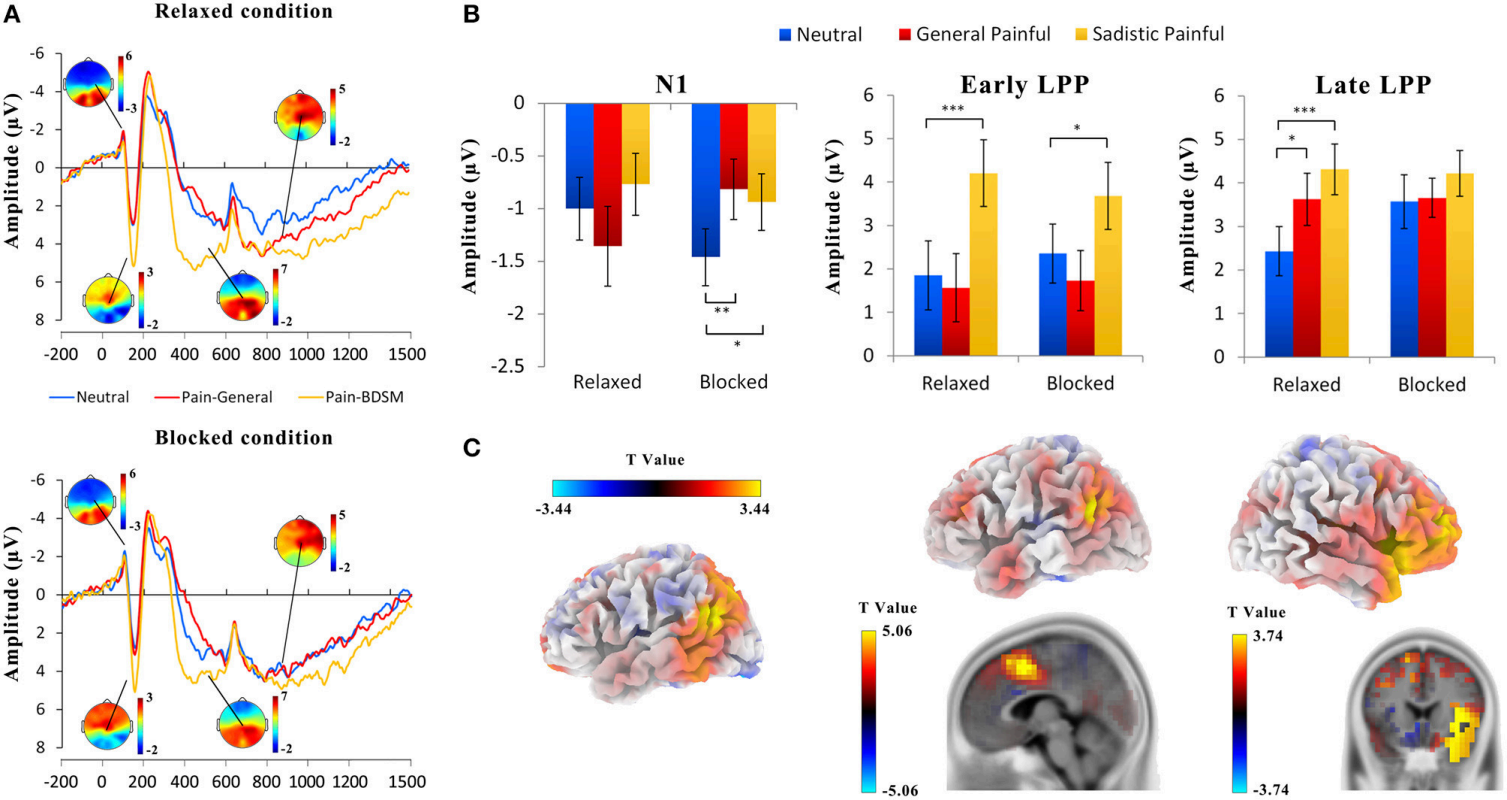
ERP results. **(A)** ERPs recorded at Cz in response to general painful, sexual sadistic painful and neutral expressions in female submissives. **(B)** The mean amplitudes at FC6 at 92–112 ms (N1), FCz at 400–600 ms (early LPP), and 700–1,000 ms (late LPP). **(C)** Illustration of the results of the source estimation. Compared with the responses to the neutral expressions, increased activities in response to the painful expressions in the N1, early LPP and late LPP time windows were identified in the left temporo-parietal junction, right superior temporal sulcus, anterior midcingulate, and right insula. ^*^p < 0.05; ^***^p < 0.01; ^***^p < 0.001.

Figure 2A illustrates the ERPs in response to the painful and neutral expressions at a frontal/central electrode. The ERPs were characterized by a negative wave at 92–112 ms (N1) and a positive deflection at 132–172 ms (P2) over the frontal/central areas, followed by a negative wave at 200–340 ms (N2) over the frontal/central region and a long LPP at 400–1,500 ms over the central/parietal area. In the present study, as in previous studies (Luo and Zhang, 2017), the LPP was evaluated as the average activity during the following two time windows: early (400–600 ms) and late (700–1,000 ms).

The ANOVAs of the N1 amplitudes at 92–112 ms over the frontal/central electrodes showed significant blocking × expression × hemisphere interactions [*F*_(2, 50)_ = 2.99~5.85, *Ps* = 0.005~0.05, η^2^ = 0.11~0.19, Figure 2B; Table 3]; the blocking × expression interactions for the N1 amplitudes were significant for the right hemisphere [*F*_(2, 50)_ = 4.50~9.53, *Ps* < 0.05, η^2^ = 0.15~0.28] but not the left hemisphere [*F*_(2, 50)_ = 1.70~2.98, *Ps* = 0.06~0.19, η^2^ = 0.06~0.11]. Separate analyses revealed significant main effects of expression under both the blocking conditions [*F*_(2, 50)_ = 4.89~7.59, *Ps* < 0.05, η^2^ = 0.16~0.23] and relaxed conditions [*F*_(2, 50)_ = 2.39~3.67, *Ps* = 0.03~0.10, η^2^ = 0.09~0.13]. The post hoc analysis further confirmed that the N1 amplitudes in response to the painful expressions in both the general and sexual sadistic contexts were lower than those in response to the neutral expressions under the blocking conditions (General painful: *Ps* < 0.01; Sadistic painful: *Ps* = 0.037~0.136) but not under the relaxed conditions (*Ps* = 0.32~0.82). These results replicated previous ERP findings (Han et al., 2016) and suggested that the early neural responses to others' suffering at frontal/central N1 were modulated by the embodied treatment. The source estimations using sLORETA suggested that the moderation effect of ball gag blocking on the neural activity during the N1 time window that differentiated between neutral expressions and painful expressions in the general contexts potentially originated in the left temporo-parietal junction and right superior temporal sulcus (peak MNI coordinates: −50, 10, −5 and 65, −45, −15; Figure 2C). To examine whether the moderation effect of the blocking condition on neural responses to others' suffering was associated with subjective feelings of the degree of facial muscle stillness, we calculated the correlations between the differential ERP amplitudes to painful versus neutral expressions between the relaxed and blocking conditions [(AmplitudeRelaxed_Pain− AmplitudeRelaxed_Neutral)− (AmplitudeBlocking_Pain− Amplitude Blocking_Neutral)] and the differential rating scores of the degree of facial muscle stillness between the relaxed and blocking conditions. The results revealed significant correlations between the subjective feelings and the blocking effect on the N1 amplitudes in response to the painful expressions in the general contexts [*r*_(26)_ = 0.38~0.58, *Ps* = 0.002~0.06] and painful expressions in the sexual sadistic contexts [*r*_(26)_ = 0.23~0.54, *Ps* = 0.005~0.26].

**Table 3:** Mean N1 amplitudes in response to each expression under the relaxed and blocked conditions.

**Electrodes**	**Expressions**	**Relaxed**		**Blocked**		**Blocking** × **Expression**		
		***M***	***SD***	***M***	***SD***	***F***	***p***	**η*p*^2^**
Fz	Neutral	−1.77	1.70	−2.15	1.64			
	General painful	−1.99	1.95	−1.73	1.56	4.341	0.02	0.15
	Sadistic painful	−1.43	1.73	−1.87	1.53			
FCz	Neutral	−1.73	1.98	−2.12	1.83			
	General painful	−1.81	2.11	−1.61	1.95	3.405	0.04	0.12
	Sadistic painful	−1.37	1.86	−1.82	1.55			
F4	Neutral	−1.65	1.72	−2.08	1.61			
	General painful	−1.92	2.01	−1.48	1.50	6.372	0.003	0.20
	Sadistic painful	−1.35	1.70	−1.70	1.45			
FC4	Neutral	−1.43	1.91	−1.87	1.70			
	General painful	−1.58	2.12	−1.20	1.72	4.498	0.02	0.15
	Sadistic painful	−1.04	1.72	−1.33	1.56			
F6	Neutral	−1.44	1.42	−1.99	1.63			
	General painful	−1.76	1.83	−1.21	1.32	9.534	<0.001	0.28
	Sadistic painful	−1.13	1.41	−1.53	1.38			
FC6	Neutral	−1.00	1.52	−1.46	1.38			
	General painful	−1.36	1.93	−0.82	1.46	7.594	0.001	0.23
	Sadistic painful	−0.77	1.49	−0.94	1.36			

The ANOVAs of the P2 amplitudes at 132–172 ms over the frontal/central electrodes showed significant main effects of expression [*F*_(2, 50)_ = 31.64~35.53, *Ps* < 0.001, η^2^ = 0.56~0.59]. The post hoc analysis revealed that compared with the neutral and painful expressions in the general contexts, the painful expressions in the sexual sadistic contexts resulted in enhanced P2 amplitudes under both the relaxed and blocking conditions (*Ps* < 0.01) The blocking × expression interactions in the P2 amplitude were not significant [*F*_(2, 50)_ = 0.44~1.21, *Ps* = 0.30~0.65, η^2^ = 0.02~0.05]. These results are consistent with previous studies (Luo and Zhang, 2017) and suggest that compared to neutral expressions, painful expressions in general contexts do not enhance the P2 amplitude in female submissives.

The ANOVAs of the early LPP amplitudes at 400–600 ms over the frontal/central electrodes showed significant main effects of expression [*F*_(2, 50)_ = 21.68~28.43, *ps* < 0.001, η^2^ = 0.46~0.53] as the amplitude of the early LPP in response to the painful expressions in the sexual sadistic contexts was larger than that in response to the neutral expressions (Figure 2B; Table 4). Interestingly, these effects were quantified by significant blocking × expression interactions [*F*_(2, 50)_ = 3.14~5.68, *Ps* ≤ 0.05, η^2^ = 0.11~0.19]. Separate analyses revealed significant main effects of expression under both conditions [relaxed: *F*_(2, 50)_ = 11.59~41.27, *Ps* < 0.001, η^2^ = 0.32~0.62; blocking: [*F*_(2, 50)_ = 6.14~17.35, *Ps* < 0.05, η^2^ = 0.20~0.41]. The *post hoc* analyses further revealed that compared with the neutral expressions, the painful expressions in the sexual sadistic contexts resulted in enhanced early LPP amplitudes under the relaxed conditions (*Ps* < 0.01). These differences were smaller under the blocking conditions (*Ps* = 0.005~0.31). There were no significant differences between the neutral expressions and painful expressions in the general contexts under both conditions (*Ps* = 0.10~1.00). The source estimations using sLORETA suggested that the moderation effect of ball gag blocking had potential sources in the anterior midcingulate cortex (peak MNI coordinates: 5, 10, 45 and −50, −50, 25; Figure 2C). The regression analyses also showed that the blocking moderation effect on the early LPP amplitudes was associated with the subjective feelings of the degree of facial muscle stillness [*r*_(26)_ = 0.60~0.74, *ps* < 0.01].

**Table 4:** Mean early LPP amplitudes in response to each expression under the relaxed and blocked conditions.

**Electrodes**	**Expressions**	**Relaxed**		**Blocked**		**Blocking** × **Expression**		
		***M***	***SD***	***M***	***SD***	***F***	***p***	**η*p*^2^**
FCz	Neutral	1.85	4.04	2.36	3.46			
	General painful	1.57	4.01	1.73	3.53	3.142	0.05	0.11
	Sadistic painful	4.21	3.91	3.68	3.93			
Cz	Neutral	3.62	4.15	3.93	3.41			
	General painful	3.67	4.18	3.39	3.70	3.453	0.04	0.12
	Sadistic painful	6.79	3.69	5.97	3.37			
FC1	Neutral	1.47	3.71	2.27	3.11			
	General painful	1.39	3.82	1.76	3.68			
	Sadistic painful	4.19	3.86	3.89	4.04			
FC2	Neutral	3.40	3.64	3.82	2.91	3.192	0.05	0.11
	General painful	3.58	3.53	3.44	3.31			
	Sadistic painful	7.05	3.60	5.85	3.66			
C1	Neutral	2.07	4.01	2.65	3.35			
	General painful	2.06	3.97	2.09	3.45			
	Sadistic painful	3.77	3.63	3.50	3.57			
C2	Neutral	3.04	4.45	3.55	3.59	5.679	0.006	0.19
	General painful	3.32	4.70	3.12	3.98			
	Sadistic painful	5.94	4.16	5.31	3.88			

**Figure 3:**
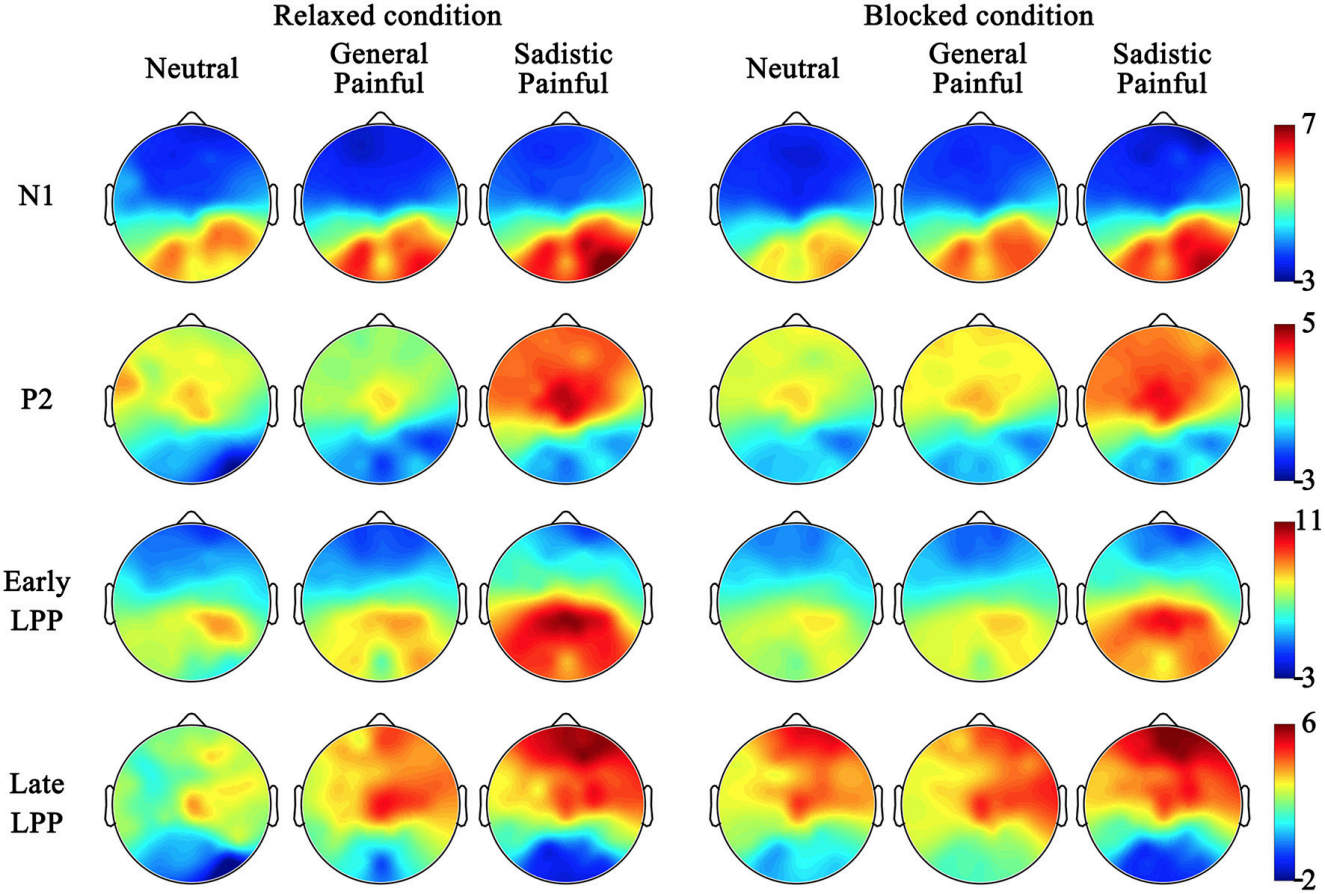
Scalp topographies for neutral, general painful and sadistic painful condition in each component.

The ANOVAs of the late LPP amplitudes at 700–1,000 ms showed significant main effects of expression [*F*_(2, 50)_ = 7.39~14.57, *ps* < 0.01, η^2^ = 0.23~0.37] because the amplitude of the late LPP in response to the painful expressions was larger than that in response to the neutral expressions (Figure 2B; Table 5). These effects were quantified by significant blocking × expression interactions [*F*_(2, 50)_ = 3.92~6.55, *Ps* < 0.05, η^2^ = 0.14~0.21]. Separate analyses revealed significant main effects of expression under the relaxed conditions [*F*_(2, 50)_ = 7.46~12.85, *Ps* ≤ 0.001, η^2^ = 0.23~0.34] but not under the blocking conditions [*F*_(2, 50)_ = 0.04~3.87, *Ps* = 0.03~0.96, η^2^ = 0.002~0.13]. The *post hoc* analyses further revealed that compared with the neutral expressions under the relaxed conditions, the painful expressions in both the general and sexual sadistic contexts resulted in enhanced late LPP amplitudes (*Ps* ≤ 0.05). There were no significant differences between the neutral and painful expressions in the two contexts under the blocking conditions (*Ps* = 0.08~1.00). The source estimations using sLORETA suggested that the moderation effect of ball gag blocking had potential sources in the right anterior insula (peak MNI coordinates: 35,20,5; Figure 2C). The blocking moderation effect on the late LPP amplitudes was not associated with subjective feelings of the degree of facial muscle stillness [*r*_(26)_ = 0.12~0.31, *ps* = 0.12~0.58] but was significantly associated with subjective feelings of humiliation [*r*_(26)_ = 0.37~0.58, *ps* = 0.002~0.06]. Sexual arousal under the ball gag blocking conditions was not significantly associated with the N1 and LPP effects. The scalp topographies for each component were provided in Figure 3.

**Table 5:** Mean late LPP amplitudes in response to each expression under the relaxed and blocked conditions.

**Electrodes**	**Expressions**	**Relaxed**		**Blocked**		**Blocking** × **Expression**		
		***M***	***SD***	***M***	***SD***	***F***	***p***	**η*p*^2^**
FC1	Neutral	1.86	2.71	3.29	2.93			
	General painful	3.24	2.82	3.29	2.34			
	Sadistic painful	3.93	2.76	4.02	2.50			
FC2	Neutral	2.49	2.84	3.74	3.10	5.513	0.007	0.18
	General painful	3.88	3.15	3.82	2.55			
	Sadistic painful	4.19	2.96	4.32	2.39			
FC3	Neutral	1.25	2.20	3.19	2.81			
	General painful	2.69	2.79	3.06	2.32			
	Sadistic painful	3.07	2.68	3.70	2.87			
FC4	Neutral	2.74	2.71	4.00	3.02	5.430	0.007	0.18
	General painful	3.79	3.52	3.96	2.60			
	Sadistic painful	5.00	2.64	4.73	2.45			
C1	Neutral	2.17	2.43	3.17	2.02			
	General painful	3.66	2.18	3.72	1.89			
	Sadistic painful	3.83	2.21	3.51	2.43			
C2	Neutral	2.88	2.53	4.05	2.59	5.882	0.005	0.19
	General painful	4.40	2.71	4.13	2.18			
	Sadistic painful	4.26	2.62	4.11	2.53			
C3	Neutral	1.65	2.40	2.84	1.95			
	General painful	2.89	2.42	3.15	1.78			
	Sadistic painful	3.39	2.47	3.16	2.37			
C4	Neutral	2.62	2.39	3.77	2.46	6.547	0.003	0.21
	General painful	4.08	3.47	4.12	2.76			
	Sadistic painful	4.73	2.07	4.15	2.03			

## Discussion

The current study aimed to determine whether and how physical restriction during BDSM practice shapes humans' responses to others' suffering and identify the underlying neural correlates. Our results showed that during the use of a ball gag to prevent facial muscle movement and facial mimicry, the neural responses to others' suffering at N1, early LPP, and late LPP were inhibited compared to the responses under the relaxed condition. Furthermore, the moderation effect of blocking with a ball gag on the N1 and early LPP amplitudes was positively correlated with the subjective feelings of facial muscle stillness, and the moderation effect of blocking on the late LPP amplitudes was positively correlated with subjective feelings of humiliation.

Researchers have proposed that empathy processes could be influenced by individuals' bodily states. Embodiment plays an important role in processing emotional information. This process is facilitated when individuals' bodily expressions of emotion are consistent with others' emotional states and is impaired when individuals' bodily expressions are inconsistent with others' emotional states (Niedenthal et al., 2001). According to this theory, early research found that asking participants to hold a pen with their teeth and lips to block the movement of expression-relevant facial muscles significantly impaired the detection of facial expressions (Niedenthal et al., 2001; Oberman et al., 2007). The manipulation of facial muscles related to pain by holding a pen horizontally using both teeth and lips modulated the empathic responses at 100–120 ms (N1) (Han et al., 2016). Consistently, our results revealed that the brain responses to both general painful and sexual sadistic painful stimuli in N1 under the blocking condition were reliably lower than those under the relaxed condition in female submissives. The subjective ratings of the degree of facial muscle stiffness confirmed that the muscle tension was stronger when the participants held a ball gag in their mouths than when they were able to move their facial muscles freely, and these subjective ratings of facial muscle stiffness were correlated with the reduced brain responses in the N1 amplitudes. These results suggest that the early sensory responses were modulated by the ball gag manipulation independent of the sexual context. Our source estimation of the moderation effect of ball gag blocking on the neural activity in the N1 latency range suggests that the mirror neuron system was engaged and that the theory of mind network (superior temporal sulcus and temporo-parietal junction) should be further examined in future neuroimaging studies.

Moreover, the neural responses of the early LPP component to the sexual sadistic painful stimuli, but not the general painful stimuli, were also lower under the blocking condition than those under the relaxed condition in the female submissives. The early LPP amplitude is sensitive to both the stimulus affective properties and the affective context (Schupp et al., 2000; Fan and Han, 2008; Olofsson et al., 2008; Dennis and Hajcak, 2009; Cacioppo et al., 2010; Decety et al., 2010). Sessa et al. (2014) found that painful contexts, but not painful expressions, selectively modulated early LPP responses when the contexts and expressions were presented. Consistent with these studies, the painful expressions in the sexual sadistic context induced larger early LPP amplitudes than the painful expressions in the general context in our study. Moreover, compared with the responses under the relaxed condition, these sadistic context-dependent early LPP responses were inhibited under the blocking condition. The source estimation of the moderation effect of ball gag blocking on the neural activity in the N1 component suggested that both the mirror neuron system (superior temporal sulcus and temporo-parietal junction) and affective network (anterior midcingulate cortex) were engaged. These results suggest that the ball gag manipulation affected not only the context-independent early sensory neural responses but also the context-dependent late neural responses. Thus, preventing facial muscle movement and facial mimicry could lead to a weaker sensitivity to sexual sadistic contexts when perceiving others' suffering.

In addition to preventing facial muscle movement and facial mimicry, when combined with other physical restraints, wearing a ball gag during BDSM practice may be related to humiliation, dehumanization (pony or animal play) or objectification (becoming an “inanimate object,” such as a foot stool) by rendering the participant unable to speak during the BDSM activities (Alison et al., 2001; Sandnabba et al., 2002; Connolly, 2006; Holvoet et al., 2017). Our results showed that the moderation effect of ball gag blocking on the late LPP amplitudes was associated with subjective feelings of humiliation, and the source estimation of the moderation effect of blocking on the neural activity in the late LPP time window suggested that the anterior insula was engaged. The insula has been suggested to play a central role in the perception of the subjective emotional state (Wager and Barrett, 2017) and the perception and awareness of multiple aspects of one's body (Craig, 2002, 2009); the representation of the interoceptive body in the anterior insula is essential for subjective feelings of the sentient self (Craig, 2010). Therefore, humiliation or dehumanization may explain the moderation effect of the ball gag on the late LPP; however, this hypothesis still needs to be investigated in future studies.

Both behavioral tendency and empathic neural response differences were found between BDSM practitioners and non-BDSM practitioners (Luo and Zhang, 2017). We reported the embodiment moderation effect on neural responses in non-BDSM practitioners in previous studies (Han et al., 2016). Thus, in this paper, we mainly focus on the embodiment moderation effect on neural responses in BDSM practitioners and did not recruit non-BDSM practitioners. Future studies may further investigate whether the humiliation effect found in this study is specific to BDSM practitioners.

Besides, some studies suggested that the pen-in-the-mouth paradigm may not be so robust. Here we want to highlight that although our paradigm seems similar to the pen-in-the-mouth paradigm, however, the logic underlying our study is quite different from that in Strack et al. (1988). In his research, Strack suggested that when participants made smile expressions, they would experience more positive emotions, which was one psychological interpret aim to oneself. However, in our research, we suggest that the experiment condition would induce muscle intension which prevent the neuron from mimicry to others' emotions and furthermore impair their empathy to other, which is one physical reaction aim to others.

Finally, given that BDSM is associated with giving and receiving pain, this study mainly focused on the effect of empathy for pain. Future studies should further investigate whether these embodiment modulation effects also occur under other negative and/or positive conditions. For example, BDSM-related stimuli may induce sexual arousal and pleasure feelings. Previous study showed that erotic pictures evoked enhanced neural responses compared with other pictures at both early (P2/N2) and late (P3/positive slow wave) temporal stages. However, in the current study, subjective sexual arousal feelings were not associated with the N1 and LPP amplitudes, these difference may cause by different task between previous and current study. Future studies could further investigate whether and how these arousal and pleasure feelings affect individuals' cognitive processes and behavior tendencies. In addition, our results suggested a “more than embodied effect” of ball gag modulation on female submissives' neural responses to others' suffering. The N1 amplitude effect in this study was similar to the effect observed in people without BDSM experience (Han et al., 2016) and was associated with the subjective feelings of facial muscle stillness. In contrast, the late LPP effect was specific to female submissives and associated with subjective feelings of humiliation. Thus, future studies should further investigate whether these humiliation-associated late LPP effects also occur in male submissives and other BDSM subgroups. Finally, future studies should further characterize the ability to empathize with others and the ability of BDSM practitioners to down-regulate their empathy.

## Conclusions

While interest in the identification of the psychological consequences of BDSM practices has increased, our EEG findings cast new light on the effect of BDSM activities on humans' responses to others' suffering by providing evidence showing that physical restriction during BDSM practices (wearing a ball gag) affects individuals' neural responses to others' suffering in both sexual sadistic contexts and general contexts. This study provides the first evidence that the transient treatment during BDSM practices affects individuals' emotional states/behavioral tendencies. Our results suggest that the use of a ball gag increases the wearer's sense of facial muscle stillness and feelings of humiliation and then inhibits the early context-independent sensory neural responses and the late sexual sadistic context-dependent neural responses. Future research should aim to further clarify whether and how various activities (e.g., bondage and roleplaying) during BDSM practices shape the perceptions of others' feelings in individuals who play different roles during BDSM practice.

## Data availability

The data generated and/or analyzed during the current study are available from the corresponding author upon reasonable request.

## Author contributions

SL designed the study. SL collected and analyzed the behavior and EEG data. SL and XZ wrote the manuscript. All authors commented on the manuscript.

### Conflict of interest statement

The authors declare that the research was conducted in the absence of any commercial or financial relationships that could be construed as a potential conflict of interest.
