# Supplementary material for: Embodiment and Humiliation Moderation of Neural Responses to Others' Suffering in Female Submissive BDSM Practitioners
Source: Front Neurosci. 2018 Jul 9;12:463. doi: 10.3389/fnins.2018.00463 (PMC6046371; doi:10.3389/fnins.2018.00463)
Supplement: Supplementary file 1 [file Data_Sheet_1.docx]

**Supplementary information:**

To further exclude the confounding factors of short prestimulus interval and low frequency noise, we also used 500ms as the prestimulus interval and a band-pass filter (1-40 Hz) to analyse our data. Change on prestimulus interval and filter did not significantly affect our results in N1 component. The ANOVAs of the N1 amplitudes at 92-112 ms over the right frontal/central electrodes showed significant blocking × expression interactions (Fz, FCz, F4, F6, FC4, FC6: F(2,50)=6.12~18.06, Ps<0.01, η2=0.20~0.42). The post hoc analysis further confirmed that the N1 amplitudes in response to the painful expressions in the general contexts were lower than those in response to the neutral expressions under the blocking conditions (Ps<0.01) but not under the relaxed conditions (Ps>0.90).

The ANOVAs of the P2 amplitudes at 132-172 ms over the frontal/central electrodes showed significant main effects of expression (F(2,50)=5.96~9.43, Ps<0.01, η2=0.19~0.27). The post hoc analysis revealed that compared with the neutral and painful expressions in the general contexts, the painful expressions in the sexual sadistic contexts resulted in enhanced P2 amplitudes under both the relaxed and blocking conditions (Ps<0.01) The blocking × expression interactions in the P2 amplitude were not significant (F(2,50)=0.004~0.30, Ps>0.70, η2=0.00~0.01).
